# The impact of conjugation strategies and linker density on the performance of the Spermine-AcDex nanoparticle–splenocyte conjugate

**DOI:** 10.1039/d5cb00104h

**Published:** 2025-07-30

**Authors:** Yuchen Su, Ruoyu Cheng, Bowei Du, Mai O. Soliman, Hongbo Zhang, Shiqi Wang

**Affiliations:** a Drug Research Program, Division of Pharmaceutical Chemistry and Technology, Faculty of Pharmacy, University of Helsinki FI-00014 Helsinki Finland shiqi.wang@helsinki.fi; b Department of Orthopaedics, Shanghai Key Laboratory for Prevention and Treatment of Bone and Joint Diseases, Shanghai Institute of Traumatology and Orthopaedics, Ruijin Hospital Shanghai Jiao Tong University School of Medicine 197 Ruijin 2nd Road Shanghai 200025 China; c Beijing Laboratory of Biomedical Materials, Key Laboratory of Biomedical Materials of Natural Macromolecules (Beijing University of Chemical Technology), Ministry of Education, Beijing University of Chemical Technology Beijing 100029 People's Republic of China; d Department of Pharmaceutics, Faculty of Pharmacy, Alexandria University Alexandria Egypt; e Pharmaceutical Sciences Laboratory, Faculty of Science and Engineering, Åbo Akademi University Biocity (3rd fl.), Tykistökatu 6A 20520 Turku Finland; f Turku Bioscience Centre, University of Turku and Åbo Akademi University Biocity (5th fl.), Tykistökatu 6A 20520 Turku Finland

## Abstract

A common approach in living medicine engineering is modifying cell surfaces with nanomedicines to form nanoparticle–cell conjugates. Despite various available strategies, limited research has examined how conjugation strategies affect the efficiency and stability of the delivery systems. Herein, we prepared polymeric nanoparticles (NPs) with protein payloads and modified them with different linkers. These NPs were conjugated to primary splenocytes using either covalent or electrostatic interactions, followed by flow cytometry analysis to evaluate the conjugating efficiency and stability. The results demonstrated that electrostatic interactions were more effective in achieving conjugation, whereas covalent interactions provided greater stability. Furthermore, the linker density on the nanoparticle surface also affected the stability. After three days of *in vitro* culture, NPs with fewer linkers were predominantly internalized by the splenocytes, whereas those with more linkers partially remained on the cell surface. Overall, this study provides fundamental insights into nanoparticle–cell conjugation, thereby contributing to living medicine design and engineering for therapeutic applications.

## Abstract 2

A common approach in living medicine engineering is modifying cell surfaces with nanomedicines. The choice of conjugation strategy and the chemical structure of the linker are crucial for living medicine design.

## Graphical abstract

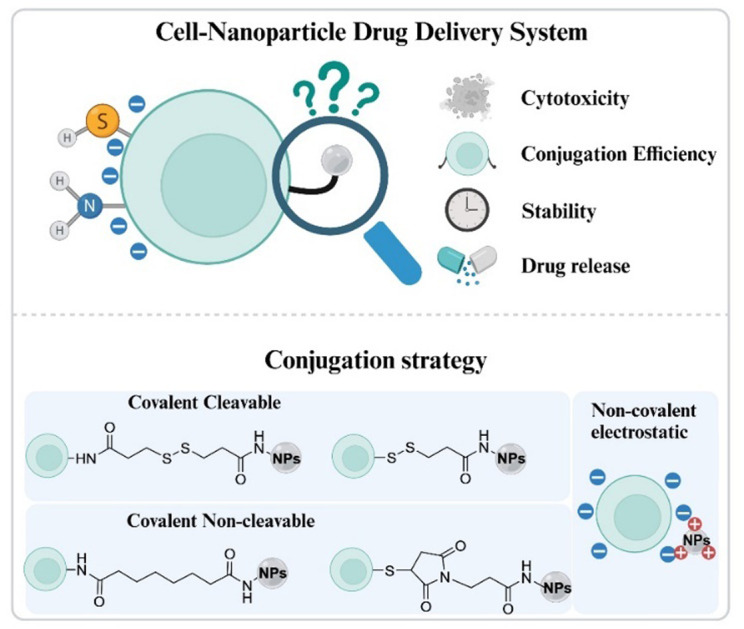

## Introduction

Combining nanomedicines with living medicines has emerged as a promising strategy for next-generation living medicine delivery.^1^ Specifically, therapeutic reagent-loaded nanoparticles (NPs) can be conjugated on the surface of living cells or internalized into the carrier cells to construct nanoparticle–cell conjugates (NCCs).^2^ By taking advantages of both nanomedicines and living medicines, the pharmacokinetic and pharmacodynamic properties, biodistribution, stability, and side effects of the payload can be precisely regulated with improved therapeutic effects in many diseases, such as cancer, multiple sclerosis, and cardiovascular disease.^3,4^ For example, in cancer immunotherapy, therapeutic nanoparticles can be conjugated to T cells to exploit their tumor-homing ability and enable localized drug release. Such NCCs have shown enhanced intratumoral T cell expansion, improved antitumor efficacy, and reduced systemic toxicity compared to conventional systemic drug administration.^5–7^

Surficial modification is a common strategy to load NPs on cell carriers. As a living carrier, the cell surface is highly heterogeneous and dynamic, consisting of lipids, proteins, and carbohydrates that are negatively charged. Therefore, cationic NPs can be easily and effectively modified on the anionic cell surface in a short time under mild conditions *via* electrostatic interactions.^8–10^ Alternatively, NPs can also be covalently conjugated to cell carriers. Specifically, the primary amine groups from protein lysine residues and the N-terminus of polypeptide chains have been widely used for nanoparticle conjugation due to the abundance and mild reaction conditions. Typically, NPs are activated by *N*-hydroxysuccinimide (NHS) ester or sulfo-NHS ester, which form amide bonds after the conjugation.^11–13^ Besides amine groups, free thiol groups from the cysteine residues are also widely used, which can react with maleimide- or dithiopyridyl-modified NPs, forming thioether or disulfide bonds.^14–16^ Additionally, metabolic labelling and click chemistry also enable covalent nanoparticle–cell conjugation by introducing unnatural reactive sites and more specificity.^17–19^

Although NCCs can be constructed through both electrostatic and covalent interactions, the conjugating efficiency and stability of NCCs may differ.^20^ Theoretically, NCCs fabricated through electrostatic interactions are affected by the abundance of negatively charged glycans on the cell surface, while the NCCs constructed through covalent interactions are influenced by the reaction efficiency and the available primary amine and thiol groups.^11,21,22^ After conjugation, the NPs attached to the cell surface may be endocytosed through dynamic membrane trafficking and degraded eventually. This process further complicates the final NCC composition. A previous study by Thomsen *et al.* reported nanoparticle immobilization on two T lymphocyte cell lines *via* seven different approaches including covalent active ester–amine, azide–alkyne cycloaddition, thiol–maleimide coupling, and non-covalent interactions. Their results confirmed that the conjugation efficiency is predominantly determined by the strategy, as well as the cell line.^23^ However, the duration for which NPs remain associated with the cells is not well understood, which is crucial for drug delivery applications. Due to the lack of comparative studies, determining the optimal conjugation strategy with desirable efficiency, stability and biocompatibility is still challenging.

**Scheme 1:**
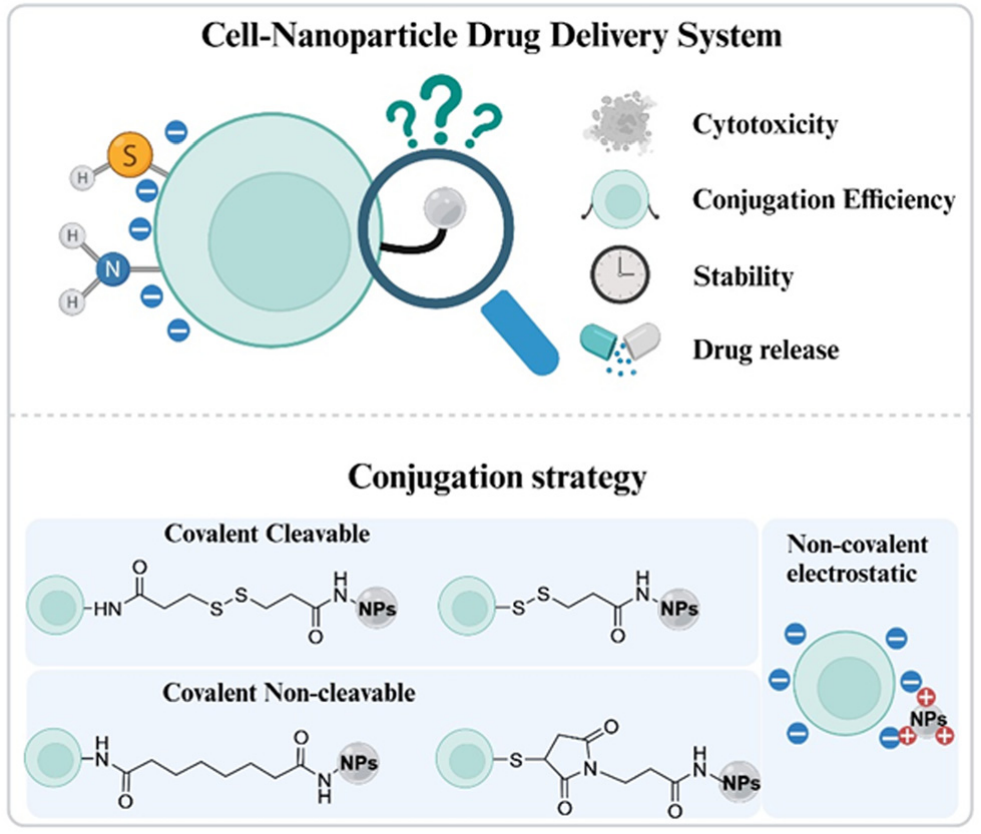
The scheme of NCC conjugation strategies *via* electrostatic or covalent interactions. Created using BioRender.com.

In this study, we aim to explore how different conjugating strategies (electrostatic and covalent interactions) and conjugating degrees would regulate the conjugating efficiency and stability of NCCs (Scheme 1). Specifically, we used cationic polymeric NPs loaded with bovine serum albumin (BSA) as model protein payloads. The cationic polymers enabled strong electrostatic interactions and provided primary amine groups for covalent conjugations. Then, we chose four different linkers, succinimidyl 3-(2-pyridyldithio) propionate (SPDP), 3,3′-dithiobis (sulfosuccinimidyl propionate) (DTSSP), (*N*-β-maleimidopropyl-oxysuccinimide ester) (BMPS) and disuccinimidyl suberate (DSS). All linkers bear NHS ester groups, enabling coupling reactions with NPs at different degrees by adjusting the linker and nanoparticle ratio. The other reactive groups of these linkers vary from maleimide and 2-pyridyldithio to NHS and sulfo-NHS, reacting with thiol or amine, respectively (Scheme 1). Two linkers (SPDP and DTSSP) have cleavable disulfide bonds after conjugation, while the others (BMPS and DSS) do not. To construct NCCs, we chose primary cells instead of cell lines, because primary cells are better mimics of real physiological conditions, and most importantly, the surface of primary cells is significantly different from that of cell lines, which is a determinant factor in conjugation strategy investigation.^24^ Specifically, we used splenocytes isolated from the spleen of C57BL/6 mice, which is commonly recognized as a T cell source in cell engineering and cancer immunotherapy.^25,26^ In total, nine types of NPs were conjugated with splenocytes *via* electrostatic, or covalent, or hybrid interactions (a combination of electrostatic and covalent interactions). The NCCs were analyzed by flow cytometry to evaluate the conjugation efficiency and further cultured for 3 days to investigate the stability *in vitro*, as shown in Scheme 1.

## Results and discussion

**Fig. 1:**
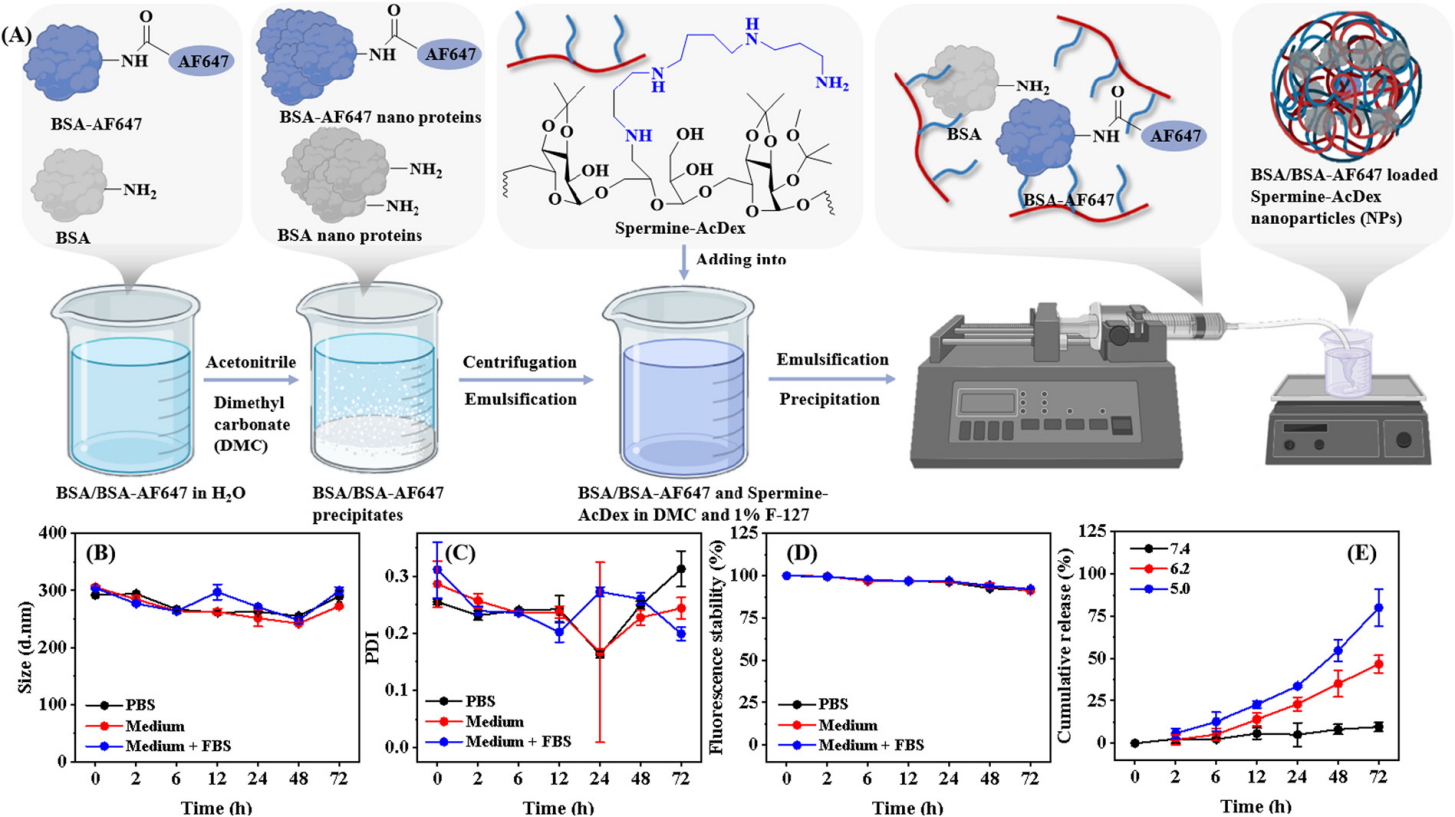
The preparation and characteristics of BSA-loaded NPs. (A) The scheme of nanoparticle preparation. (B–D) Size, PDI, and fluorescence intensity in different media over 3 days. (E) The release behavior of NPs at different pH values. Mean ± SD (*n* = 3).

To fabricate NCCs, we first prepared positively charged polymeric NPs, using spermine-modified acetalated dextran (Spermine-AcDex).^27^ The chemical structure of Spermine-AcDex was determined by nuclear magnetic resonance (NMR) spectroscopy, as shown in Fig. S1, which is consistent with the results of other researchers.^19^ We chose Spermine-AcDex because these NPs can load protein drugs with ultrahigh encapsulation efficiency.^28^ Considering the fact that each cell can carry only a very limited number of NPs, the ultrahigh drug loading capacity of these NPs maximizes the overall drug loading in the NCCs and thus enhances the therapeutic potential. Furthermore, Spermine-AcDex NPs have abundant amine groups on the surface, providing reactive sites for covalent modification and positive charges for the potential electrostatic interactions (Fig. 1A).

Then, we used BSA as the model payload to fabricate protein loaded NPs following literature reports.^29^ Briefly, the fabrication of NPs started with the precipitation of the payload, followed by the emulsification with the Spermine-AcDex polymer, the solvent diffusion and solidification processes (Fig. 1A). We also used Alexa Fluor 647 labelled BSA (BSA-AF647) in the encapsulation because fluorescence labelling makes it possible to characterize the nanoparticle conjugation efficiency, stability and drug release in the following experiments. The size of BSA-AF647 encapsulated NPs was 284.7 ± 0.4 nm, characterized by dynamic light scattering, with a polydispersity index (PDI) of 0.17 ± 0.02 (Fig. S2A). The zeta potential of NPs was 33.7 ± 0.4 mV (Fig. S2B). Additionally, the morphology of NPs was round, as shown in the transmission electron microscopy (TEM) images (Fig. S2C). The NPs showed good stability in phosphate-buffered saline (PBS), culture medium (Roswell Park Memorial Institute, RPMI 1640), and RPMI 1640 supplied with 10% fetal bovine serum (FBS) for up to 3 days without significant changes in the size and PDI (Fig. 1B and C). Additionally, the fluorescence intensity of NPs was also stable under different culture conditions, revealing that the payload (BSA-AF647) was still inside the NPs (Fig. 1D). Then, we further explored whether the payload would be released under the acidic environment since the acetal groups of Spermine-AcDex are prone to hydrolysis.^30–32^ Therefore, the release behavior of NPs was evaluated at different pH values (7.4, 6.2, and 5.0). Up to 3 days, limited release was observed at pH 7.4 (Fig. 1E), which was consistent with the results of fluorescence stability. In contrast, significant payload release was observed at pH 6.2 and 5.0. Furthermore, as the pH decreased, the release accelerated due to the accelerated degradation of NPs. The favorable stability and pH-responsiveness suggested NPs as a suitable candidate for the construction of NCCs in cancer therapy due to the acidic tumor microenvironment.^33^

**Fig. 2:**
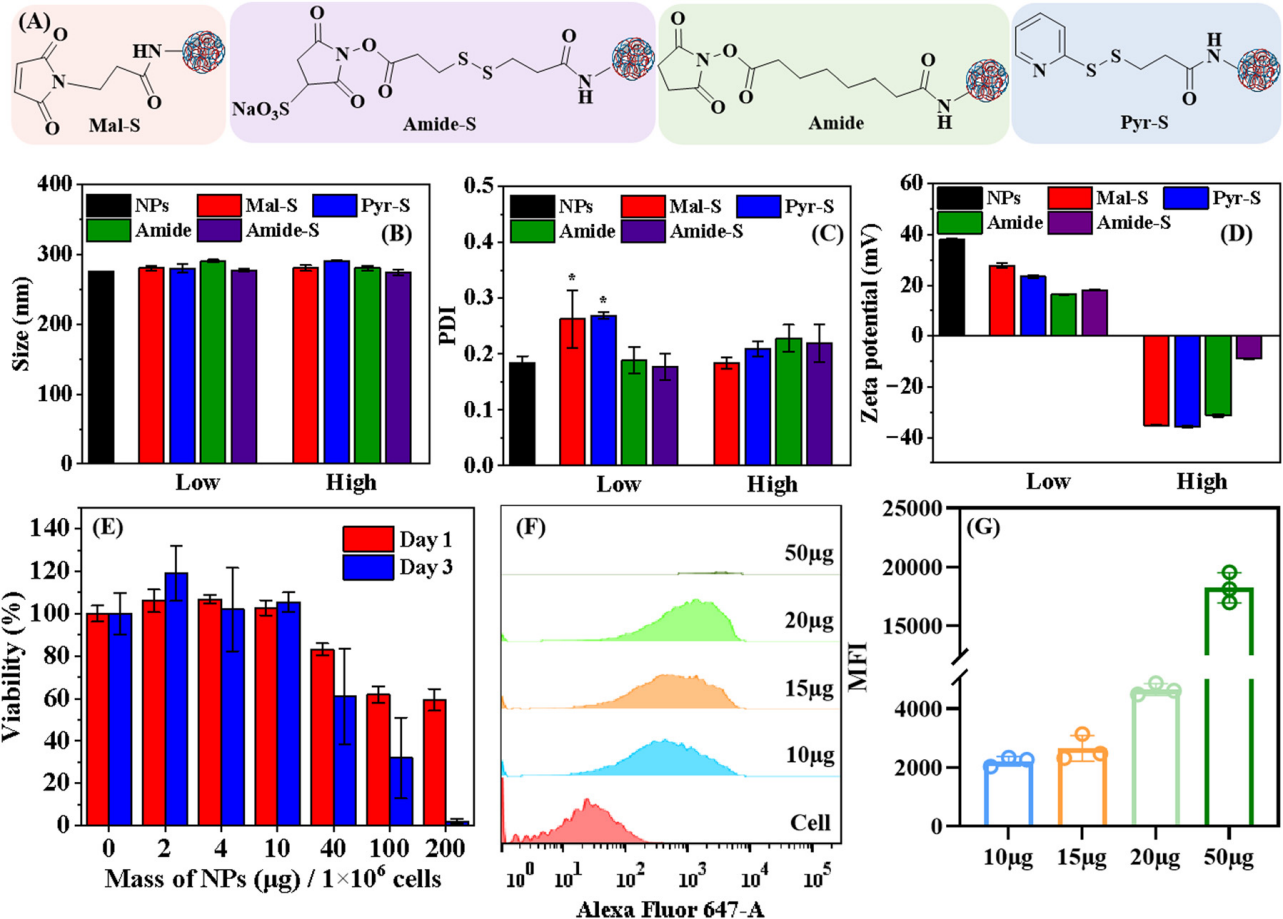
The physiochemical properties of NPs modified with BMPS, DTSSP, DSS, and SPDP. (A) The scheme of NPs modified with BMPS (Mal-S), DTSSP (Amide-S), DSS (Amide), and (SPDP) Pyr-S. Results of Mal-S, Amide-S, Amide, and Pyr-S with a high or low degree of modification on (B) size, (C) PDI, and (D) zeta potential. (E) The cell viability of splenocytes incubated with different amounts of NPs. (F) The flow cytometry histograms and (G) the mean fluorescence intensity (MFI) of splenocytes conjugated with different amounts of NPs. Mean ± SD (*n* = 3). For C, data were analyzed by the one-way ANOVA test, * *P* < 0.05.

After investigating the properties of NPs, we conjugated linkers onto NPs before linking to cells. Elemental analysis was used to determine the number of amine groups for linker conjugation. As shown in Table S1, 0.91 ± 0.02% N was determined in Spermine-AcDex, indicating that 2 mg of NPs contained approximately 0.28 μmol amine groups. Four different linkers BMPS, DTSSP, DSS, and SPDP (the chemical structures are shown in Fig. S3) were chosen in this study. The variation of reactive groups and the presence of disulfide are hypothesized to affect the conjugation efficiency, stability and the final NCC performance in the following studies. First, we explored whether the conjugation of linkers on the surface of NPs would change the physiochemical properties of NPs, *i.e.*, the size, PDI, and zeta potential. The NPs modified with BMPS, DTSSP, DSS, and SPDP were denoted as Mal-S, Amide-S, Amide, and Pyr-S, respectively, in the following text, as shown in Fig. 2A. For each linker, we also explored different ratios between amine groups on the NPs and NHS ester linkers in the conjugation, because a higher linker amount on NPs’ surface may result in multiple covalent conjugation with cells simultaneously, further stabilizing the NPs. We tuned the molar ratio between the amine groups of NPs and the NHS ester of linkers at 1 : 1 (high-degree modification, denoted as high) and 50 : 1 (low-degree modification, denoted as low), respectively.

As shown in Fig. 2B and C, different linkers and degrees of modification had limited influences on the size and the PDI of NPs. Although the Mal-S (Low) and Pyr-S (Low) exhibited higher PDI than other groups, the PDI was still lower than 0.3, indicating an acceptable level of size homogeneity (Fig. 2C). Regarding zeta potential, all NPs with high-degree modifications had negative surficial charges (Fig. 2D). This is because after reaction with linkers, the surface amine groups of NPs were converted to amides, which do not protonate under physiological conditions. In contrast, NPs with low-degree modifications still retained their positive surficial charges, though their zeta-potential was lower than that of NPs without modifications. These results suggest that at low-degree modification, the available amine groups of NPs were reduced but not eliminated. The differences in zeta-potential of linker-modified NPs indicate that all NPs with low-degree modifications might conjugate to cells *via* both electrostatic and covalent interactions. In contrast, covalent interactions would be the dominant force for constructing NCCs with NPs with high-degree modification, since both cell membranes and NPs are negatively charged in these cases.

After NP fabrication and linker modification, we proceeded with NPs and cell conjugation. First, we isolated splenocytes and analyzed the cell types by flow cytometry using cell-specific biomarkers, revealing that 27.0% were T cells and about 9.69% were cytotoxic T cells (Fig. S4). Then, the biocompatibility of NPs (without any surficial modification) on splenocytes was investigated *in vitro*. Different amounts of NPs (from 0 μg to 200 μg in 200 μL) were incubated with 1 × 10^6^ splenocytes for 1 and 3 days. When the amount of NPs was lower than 10 μg, no cytotoxicity was observed after 1 and 3 days of incubation. The NPs started to show toxicity when the amount was above 40 μg, which was also observed in other studies using Spermine-AcDex NPs,^34^ possibly due to the positive charge (Fig. 2E).

Based on the cytotoxicity results, we further optimized the ratio between NPs and splenocytes to balance cytocompatibility and conjugation efficiency. First, we fixed the amount splenocytes at 1 × 10^6^ and evaluated whether increased amounts (from 10 to 50 μg) of NPs would exhibit detectable fluorescence *in vitro*. The gating strategy for flow cytometry is shown in Fig. S5. For all flow cytometry experiments, we first gated the main cell population based on forward and side scatter (FSC-SSC) profiles (Fig. S5, left panel) and then analyzed the Alexa Fluor 647 signal within this gated population (Fig. S5, right panel). Fig. S5 shows the baseline fluorescence from the negative control, which we used to define the threshold for Alexa Fluor 647-positive events (set at fluorescence intensity >10^2^). This gating strategy was applied consistently across all samples.

As shown in Fig. 2F and G, the positive event percentage increased from 72.4 ± 4.6% (10 μg NPs) to 99.0 ± 1.2% (50 μg NPs), and the MFI of all NP-conjugated samples significantly increased compared with that of control. However, as shown in Fig. S6, the cell population obviously changed when the splenocytes were incubated with 50 μg of NPs, indicating that the NPs’ conjugation could change the cell morphology and cell behaviors. The lack of visible signals of 50 μg of NPs in Fig. 2F was due to the minimal number of cells falling within the FSC-SSC gate. As shown Fig. S6, increasing the NP dose resulted in a progressive reduction in the gated cell population. Specifically, NPs (50 μg) only exhibited 1.43% cell population in the gate. This cell population shift is likely caused by changes in the cell morphology induced by the high NP concentration. Such morphological alterations may influence cell behavior and ultimately have a negative impact on the function of NCCs. Therefore, considering both the fluorescence intensity and cell scattering profiles of the NCCs, we selected 20 μg of NPs as the optimal condition for the following experiments.

**Fig. 3:**
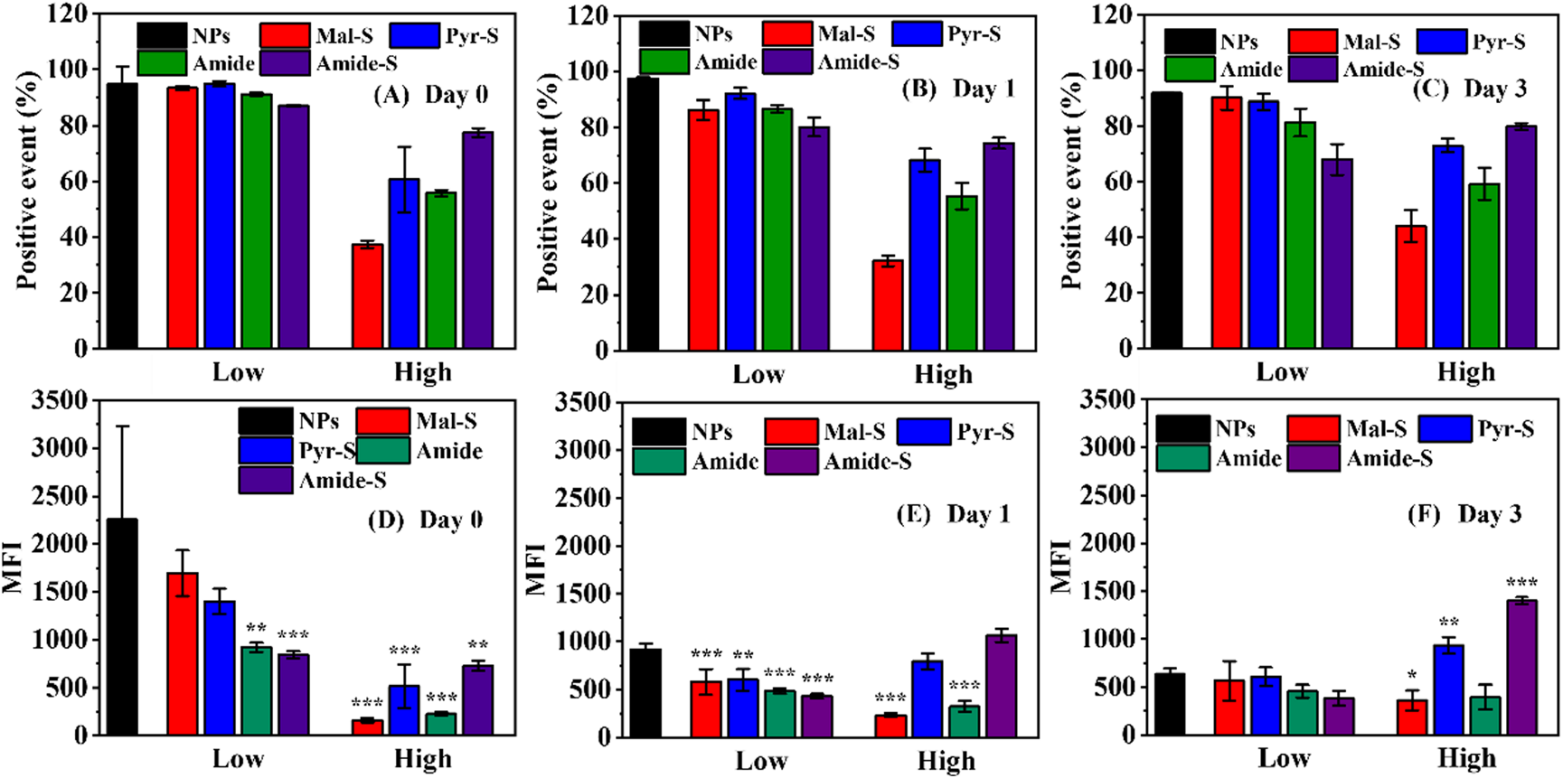
The stability of NCCs *in vitro*. Fabrication and culturing of the NCCs and then quantitative analysis of the percentage of positive events (splenocytes modified with NPs) after (A) 0 day, (B) 1 day, and (C) 3 days *in vitro* and the MFI of NCCs after (D) 0 day, (E) 1 day, and (F) 3 days *in vitro*. Mean ± SD (*n* = 3). *p < 0.05, **p < 0.01 ***P< 0.001, one-way ANOVA with Tukey's HSD to determine significance between NPs and other groups.

Then, we evaluated the conjugation efficiency and the stability of the NCCs *in vitro*, by monitoring the MFI of NCCs for 3 days. As shown in Fig. 3A–C, over 80% splenocytes were modified in NPs and low-degree modification groups for up to 3 days, which could be attributed to the successful and efficient conjugation *via* electrostatic interactions. In contrast, NPs with high-degree modifications exhibited lower conjugating efficacy (positive events ranging from 37.4 ± 1.3% to 77.5 ± 1.6%), revealing that covalent binding is less efficient compared with electrostatic interactions. Then we analyzed how the MFI changed over time. Compared with day 0 (Fig. 3D), the MFI of NCCs (NPs) on day 1 decreased by approximately 50% on day 1 (Fig. 3E). Similarly, the MFI of NCCs (Mal-S, Pyr-S, Amide, and Amide-S, Low) also decreased to around half of their day 0 levels. The decreased MFI could be attributed to the decreased amount of NPs associated with cells, possibly due to the nanoparticle detachment from the cell surface. In contrast, a limited MFI decrease was observed on NCCs (Mal-S High and Amide High), revealing the high stability. In addition, the NCCs with cleavable disulfide bonds (Pyr-S High and Amide-S High) presented higher MFI than other covalent NCCs (Mal-S and Amide, High), suggesting that the disulfide conjugating reaction was more effective than the amine-NHS ester reaction. Notably, the NCCs (Amide-S High) exhibited the highest MFI among NCCs with high-degree modifications, possibly due to the potential double conjugating strategies. The disulfide bond and NHS ester in Amide-S could react with both amine and thiol groups on the splenocyte surface, which consequently improved the conjugating efficacy of Amide-S (High).

After three days, the MFI of NCCs (NPs) further decreased compared to day 1 (Fig. 3F). However, the MFI of NCCs (Mal-S, Pyr-S, Amide, and Amide-S, Low) did not obviously drop compared to day 1, which means that the remained NPs were associated with splenocytes from day 1 to day 3 due to the covalent interaction. These results suggested that there was a hybrid interaction in NCCs (Low): the electrostatic interaction was more efficient than covalent binding, while the covalent interaction allowed for long-term stability of the NPs.

Similarly, the MFI of NCCs (Mal-S, Amide, and Pyr-S, High) was consistent with the MFI on day 1, revealing the acceptable stability of Mal-S, Amide, and Pyr-S (High) even after 3 days of *in vitro* culturing. However, the MFI of Amide-S (High) further increased compared to the MFI on day 1. Then, we assumed that the consistently increased MFI could be attributed to the internalization of NPs into splenocytes, followed by the intracellular release of the payload. The released BSA-AF647 potentially exhibited higher fluorescence intensity than the partially quenched BSA-AF647 encapsulated in NPs, which has been approved in this study (Fig. S7) and by other researchers.^35^

**Fig. 4:**
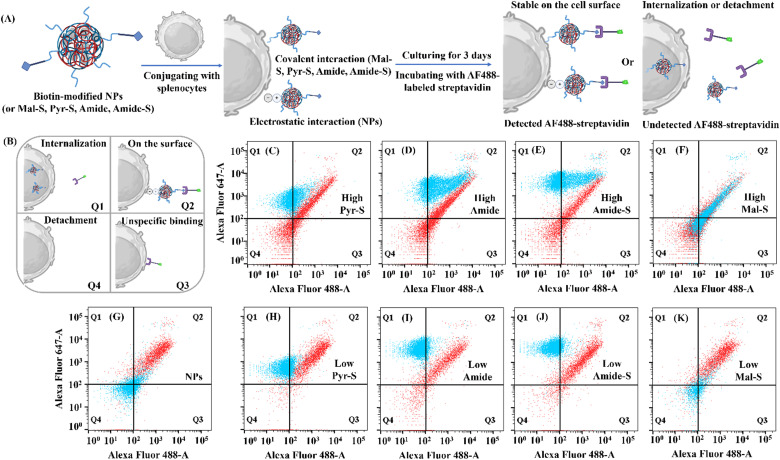
Investigation of the stability of NCCs *via* biotin–streptavidin interactions *in vitro*. (A) The scheme of probing biotinylated NCCs with AF488-streptavidin *in vitro*. (B) The scheme of different nanoparticle–cell association status in the flow cytometry results. Q1: NCCs with internalized NPs; Q2: NCCs with surface-conjugated NPs; Q3: NCCs with nonspecific streptavidin binding; and Q4: cells without NPs or streptavidin. (C)–(K) The flow cytometry results of different NCCs on day 0 (red dots) and day 3 (blue dots): (C) Pyr-S (High), (D) Amide (High), (E) Amide-S (High), (F) Mal-S (High), (G) NPs, (H) Pyr-S (Low), (I) Amide (Low), (J) Amide-S (Low) and (K) Mal-S (Low).

To validate our hypothesis and further explore whether the conjugated NPs were on the surface of splenocytes or internalized during *in vitro* culture, we used the biotin–streptavidin assay as reported in the literature.^18^ We modified all types of NPs with biotin before cell conjugation and constructed NCCs following our previous protocols (Fig. 4A). At each time point, we probed cell surface NPs by incubating Alexa Fluor 488 (AF488) labeled streptavidin with NCCs. Since the interaction between biotin and streptavidin is highly specific, we assume that this assay can detect surface-binding NPs with high sensitivity and provide quantitative insights. If the biotinylated NPs were still on the splenocyte surface, the AF488-streptavidin would bind with biotin, and then both AF647 and AF488 can be detected by the flow cytometry (Fig. 4B, Q2). If the biotinylated NPs were internalized into the splenocyte or detached from the splenocyte, then there should be minimal AF488 signal detected by flow cytometry (Fig. 4B, Q1 and Q4). Otherwise, the AF488-streptavidin may also unspecifically bind to cells, showing a limited AF488 signal without an AF647 signal (Fig. 4B, Q3).

As shown in Fig. 4C–K (red dots), on day 0, the cell populations of NCCs in all groups fall on the diagonal on the plots, suggesting that almost all of the conjugated NPs were on the surface of the cells, regardless of conjugation strategies. NCCs (NPs) had higher conjugation efficiency reflected by a high percentage of Q2 distribution (Fig. 4G), while all NCCs with high-degree linker modifications had both Q2 (double positive) and Q4 (double negative) distributions, suggesting that not all cells have NP conjugation. This is consistent with the previous results shown in Fig. 3A. In contrast, on day 3, the NCCs in different groups showed very distinct population distributions (Fig. 4C–K, blue dots). Notably, the population of NCCs (Mal-S, High) remained almost unchanged compared with day 0 (Fig. 4F), suggesting that the Mal-S was stable on the surface of splenocytes. The population of other NCCs with high-degree modification (Pyr-S, Amide, and Amide-S, High) presented on both Q1 and Q2, which indicates that some NPs had been internalized into splenocytes and some still remained on the surface. As for the NCCs with low-degree modifications (Fig. 4H–J), the main populations shifted to Q1, suggesting that almost all NPs have been internalized. Two NCCs (NPs and Mal-S, Low) showed population shifting to Q4 (Fig. 4G and K), indicating significant removal of NPs from cell carriers. These results suggest that the conjugation strategy and the linker modification degree determined the fates of the NPs after conjugation.

**Fig. 5:**
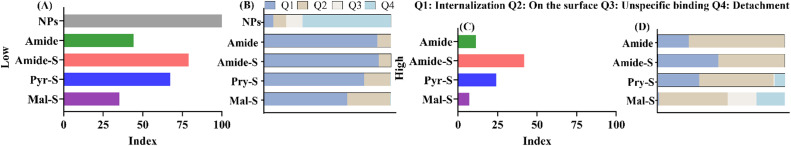
Summary of the conjugation strategies. Evaluation of the NPs with a low degree of modification in terms of (A) conjugation efficiency index and (B) stability and NPs with a high degree of modification in terms of (C) conjugation efficiency index and (D) stability.

Given the above results, we summarize the comprehensive understanding about conjugation strategies in Fig. 5. Here, we show the nanoparticle conjugation efficiency as an index (0–100), based on the Day 0 nanoparticle MFI (in Fig. 3D) normalized to the most efficient group (NPs). We also show the stability of nanoparticles on cell carriers in the stacked bar charts, based on the biotin–streptavidin assay results on Day 3 (Q1–Q4 percentage in Fig. 4B). It is clear that the non-covalent electrostatic interaction can fabricate NCCs with superior conjugating efficiency, despite the significant dissociation between cells and NPs within 3 days (Fig. 5A and B). Nevertheless, the electrostatic interaction is a convenient strategy, which involves simple mixing of cationic NPs and cell carriers without chemical modification, and thus could be a suitable choice for *in situ* co-delivery of NPs and cells.^36,37^ All NPs with low modifications (hybrid interaction) exhibited lower conjugating efficiency compared with those using electrostatic interactions, and most NPs were internalized into the host cells after 3 days (Fig. 5A and B). The nanoparticle internalization was also found in covalent conjugation strategies (Pry-S, Amide and Amide-S), albeit with a lower percentage (Fig. 5D). In these cases, the drugs encapsulated by the NPs might be gradually released into the host cell and thus are suitable for regulating the host cell behavior after NCC administration. For example, immune cells and mesenchymal stem cells have plasticity which could be reprogrammed under the regulation of nucleic acids, peptides, and cytokines.^38^ Therefore, when using immune cell based NCCs, we can apply these strategies to load therapeutics in NPs and tune the cell carrier to a certain phenotype suitable for immunomodulation in pathological conditions (cancer, inflammation, *etc.*).^39–41^ At the same time, all of the NCCs with high-degree linker modification showed a significant amount of NPs remaining on the surface (Fig. 5D). We assume that these NCCs could be suitable for delivering drugs to targeted sites and simultaneously regulating the host cell and surrounding cells.^39^ Despite the low conjugation efficiency (Fig. 5C), the great stability suggests the possibility for long-term drug release after host cells migrate to the target tissue.

## Conclusions

In summary, this study provides a systematic investigation into conjugation strategies for NCC construction. Our results reveal that the choice of conjugation strategy and the chemical structure of the linker have a significant impact on the nanoparticle loading capacity and stability on cell carriers. Importantly, these findings suggest that it is challenging to achieve high conjugation efficiency and stability at the same time. Therefore, a suitable conjugation strategy choice should be made by carefully balancing these two factors in the specific NCC application scenario. We admit that the optimal NCC strategy is cell-specific, and this study has only investigated mice splenocytes. Additionally, mice splenocytes consist of various cell types, such as B cells, T cells, dendritic cells, macrophages, and natural killer cells. The influence of each cell type on the conjugating efficiency and stability of NCCs is still unknown. Moreover, this study did not perform *in vivo* experiment, and hence the biodistribution, stability, and potential therapeutic application of NCCs are unknown *in vivo*. Nevertheless, we envision that the mechanistic insights will benefit future designs of NCCs for cell engineering, adoptive cell therapies, and site-specific drug delivery. For example, this study can potentially provide a conjugating strategy to modify cluster differentiation of the (CD)8^+^ T cell surface with anti-cancer therapeutic reagent loaded nanoparticles. Through the homing properties of CD8^+^ T cells, both CD8^+^ T cells and nanoparticles can accumulate on tumor to improve the therapeutic effects.

## Ethical statement

The experimental procedures (isolation of mice primary splenocytes) were approved by the Experimental Animal Center of the University of Helsinki (decision number: KEK23-034). All the procedures were performed in accordance with the guidelines from the animal facility of the University of Helsinki.

## Author contributions

Y. S.: data curation and analysis, methodology and investigations, and writing – original draft. R. C.: conceptualization, methodology and investigations, writing – original draft, reviewing and editing. B. D.: supporting role in investigations related to cytotoxicity and nanoparticle–cell conjugation. M. O. S.: TEM imaging. H. Z.: supporting role in supervision and project administration, reviewing and editing. S. W.: conceptualization, supervision, resources, reviewing and editing.

## Conflicts of interest

There are no conflicts to declare.

## Supplementary Material

CB-006-D5CB00104H-s001

## Data Availability

The data that support the findings of this study are available in the Zenodo repository, with the DOI: https://doi.org/10.5281/zenodo.15130329. Materials and methods, and supporting figures. See DOI: https://doi.org/10.1039/d5cb00104h
