# Supplementary material for: The impact of conjugation strategies and linker density on the performance of the Spermine-AcDex nanoparticle–splenocyte conjugate
Source: RSC Chem Biol. 2025 Jul 30;6(10):1546–54. doi: 10.1039/d5cb00104h (PMC12352615; doi:10.1039/d5cb00104h)
Supplement: CB-006-D5CB00104H-s001 [file CB-006-D5CB00104H-s001.pdf]

Supplementary Information (SI) for RSC Chemical Biology.
This journal is © The Royal Society of Chemistry 2025

Electronic supplementary information

Impact of Conjugation Strategy and Linker Density on Spermine AcDex Nanoparticle-Splenocytes Conjugates Performance

Yuchen Su,[†a] Ruoyu Cheng,[†a,b] Bowei Du,[a,c] Mai O. Soliman,[a,d] Hongbo Zhang[e,f] and Shiqi Wang[*a]

Experimental Section

1. The synthesis and characterization of spermine-modified acetalated dextran.

The spermine-modified acetalated dextran was synthesized according to the literature report.[42] Dextran (5.0 g, 30.9 mmol, molecular weight: 9000-11000 g/mol, from Leuconostoc mesenteroides, Sigma-Aldrich, USA) was dissolved in 20 mL water, followed by reacting with sodium periodate (1.1 g, 51.4 mmol, Sigma-Aldrich, USA) for 5 hours at room temperature. Then, the product was dialyzed in Milli-Q water with a regenerated cellulose membrane (molecular weight cutoff: 3500 g/mol, Spectra/Por RC tubing, USA), followed by the lyophilization to get the partial oxidized dextran (O-Dex).

The O-Dex (1.0 g) was dissolved in 10 mL anhydrous dimethyl sulfoxide (DMSO, Sigma–Aldrich, USA), followed by reacting with pyridinium p-toluenesulfonate (PPTS, 15.6 mg, Sigma–Aldrich, USA) and 2-methoxypropene (3.4 mL, Sigma–Aldrich, USA) for 4 hours under the argon atmosphere. Then, the reaction was quenched by triethylamine (TEA, 1 mL, Sigma–Aldrich, USA). The polymer product was obtained by precipitating the solution in Milli-Q water (100 mL, pH 8-9). After centrifugation (15 min, 16110 × g), the pellet was dissolved in ethanol (20 mL) and precipitated in Milli-Q water again. The purification process was repeated twice, and the residual water was removed by lyophilization for 48 hours to obtain the partially oxidized acetalated dextran (O-AcDex).

O-AcDex (2.0 g, 12.3 mmol) was stirred with spermine (4.0 g, 19.8 mmol, Sigma–Aldrich, USA) in 10 mL DMSO at 50°C for 22 hours. Then, $NaBH_4$ (2.0 g, 52.9 mmol, Sigma–Aldrich, USA) was reacted with the solution for another 18 hours at room temperature. The white product was totally dissolved in ethanol, followed by precipitating in Milli-Q water (40 mL). The product was isolated by centrifugation (10 min, 16110 ×g). The resulting pellet was washed thoroughly with Milli-Q water (pH 8). The spermine-modified acetalated dextran (Sp-AcDex) was obtained by the lyophilization. The degree of amination was quantified by the elemental analysis (HANAU Elementar Analysensysteme GmbH, Germany). Analyzes were performed in triplicates in CHNS mode using the method 2mgChem70s. Carbon was determined as CO_2, hydrogen as H_2O, nitrogen as N_2 and sulfur as SO_2. N_2 is not adsorbed in the adsorption column and is the first measuring component to enter the thermal conductivity detector (TCD). CO_2, H_2O and SO_2 are adsorbed together in the adsorption column. The adsorption column is heated stepwise to desorption temperatures of CO_2 (60 °C), H_2O (140 °C) and SO_2 (210 °C). The measured gas enters the detector with the carrier gas (He) one by one. The percentage elemental concentration of

the element in the sample is calculated using formula $c\ \% = \frac{a \cdot 100 \cdot f}{w}$, where c is the element concentration [%], a absolute element content [mg], f the daily factor and w the sample weight [mg]. The calculation of amine groups in Spermine-Acdex based on the N% was calculated as the following formulation:

—

Here, the m_{NPs} is the weight of Spermine-Acdex nanoparticles; 0.91% is the percentage of N according to the element analysis; 4 is the total number of atomic nitrogen in Spermine-Acdex molecule; 14 is the atomic mass of nitrogen. Of all four atomic nitrogens, only one is $-NH_2$ group and the other three are -NH- groups (see the molecular structure of Spermine-Acdex in Fig S1.).

2. The synthesis and characterization of Sp-AcDex NPs

Nanoparticle synthesis: The synthesis of BSA loaded Sp-AcDex NPs was performed according to literature with slight modifications.[24,25] BSA (A8806, Sigma–Aldrich, USA) was used as a model protein drug in this experiment. Alexa Fluor 647 ester (Lumiprobe, USA) was reacted with BSA (1:10, molar ratio) to label the BSA (BSA-AF647) for the following experiments. Different mass ratio between BSA and BSA-AF647 (9:1, in total 6 mg) were dissolved into 300 µL H_2O. Then, 2.7 mL acetonitrile (Sigma–Aldrich, USA) was added to the mixture of BSA and BSA-AF647 solution with constant stirring, followed by further precipitation with 9 mL dimethyl carbonate (Sigma–Aldrich, USA) and centrifuging at 5000g for 5 mins to obtain the BSA and BSA-AF647 pellet. Spermine-AcDex polymer was dissolved in dimethyl carbonate to prepare the polymer solution (20 mg/mL). Afterward, the pellet was resuspended with 600 µL polymer solution, followed by adding 1.2mL Pluronic F-127 (1%, m/v, Sigma–Aldrich, USA) solution with tip sonication (20% power, 10 seconds-on and 5 seconds-off for 30 seconds working time, Sonics VCX 750, USA). Then, 6 mL 1% Pluronic F-127 was pumped (at 15 mL/h) into the abovementioned solution with constant stirring, followed by centrifugating at 16100g for 10 mins to remove the free BSA, BSA-AF647, and 1% Pluronic F-127.

Particle size and morphology characterization: After washing with H_2O, the NPs were characterized by the dynamic light scattering (DLS, Malvern) regarding their size, PDI, and zeta potentials. TEM (JEM-3200FSC JEOL microscope) was used to analyze nanoparticle morphology. Samples were freshly prepared prior to analysis. A 5 µL drop of each sample was deposited on a carbon-coated copper grid and blotted with filter paper to remove excess liquid. The grids were left to air dry overnight before examination. Images were taken at X80k magnification at 80 kV acceleration voltage using Digital Micrograph software (Gatan). No sample pretreatment (e.g., staining, filtration) was performed.

Fluorescence stability: The NPs were incubated in different mediums (1×PBS, RPMI1640, and RPMI1640 with 10%FBS) with constant stirring at 37 °C. At each timepoint, the size, PDI, and zeta potential of NPs were investigated by the DLS. Additionally, the NPs were centrifugated (16100g

for 10 mins), and the FI of suspension was measured by the Varioskan. Then the fluorescence stability of NPs was calculated by the following equation:

$$\text{Fluorescence stability (\%)} = \frac{FI_{NPs} - FI_{Suspension}}{FI_{NPs}} \times 100\%$$

FI_{NPs}: The FI of NPs after the synthesis; $FI_{suspension}$: The FI of suspension at each time point

Drug release: 0.1 mg NPs were incubated in 1mL 1×PBS with different pH (7.4, 6.8, and 5.0). At each time point, 100 µL solution was withdrawn and centrifugated (16100g for 10 mins). Then the FI of suspension was measured by the Varioskan. Additionally, 0.1 mg NPs were hydrolyzed in 10µL hydrochloric acid (0.1M) and diluted to 100µL with 1×PBS, followed by measured by the Varioskan. The cumulative release was calculated as the following equation:

$$Cumulative\ release = \frac{FI_{Suspension}}{FI_{Control}} \times 100\%$$

$FI_{Suspension}$: The FI of suspension; $FI_{Control}$: The FI of 0.1 mg hydrolyzed NPs

The fluorescence intensity comparison of free and encapsulated BSA-AF647: 0.1 mg NPs loaded with BSA-AF647 and equal amount of BSA-AF647 (calculated by 0.1 mg NPs' loading efficiency) were dispersed or dissolved in PBS, then the fluorescence intensity of both solutions were quantified by the Varioskan.

3. The modification of NPs and characterization

After the synthesis of NPs, we modified them with different linkers, succinimidyl 3-(2-pyridyldithio) propionate (SPDP, TCI), disuccinimidyl suberate (DSS, TCI), 3,3'-dithiobis (sulfosuccinimidyl propionate) (DTSSP, TCI), and N-β-maleimidopropyl-oxysuccinimide ester (BMPS, TCI). First, these linkers were dissolved in dimethylsulfoxide (DMSO) respectively to prepare the stock solution (3.12 µg/mL SPDP, 3.68 µg/mL DSS, 4.04 µg/mL DTSSP, and 2.68 µg/mL BMPS). Then, 1 mg/mL NPs were respectively reacted with these linkers (1:9×10^{-4} for high degree of modification, 1:1.8×10^{-5} low degree of modification, molar ratio between the amine of NPs and the NHS of linkers) in the 0.1 M $NaHCO_3$ solution (Sigma–Aldrich, USA) for 20 mins with light protection to synthesize SPDP-modified NPs (Pyr-S), DSS-modified NPs (Amide), DTSSP-modified NPs (Amide-S), and BMPS-modified NPs (Mal-S) with high or low degree of modifications (denoted as High or Low). After the reaction, all NPs were centrifugated (16100g, 10 mins) and washed with H_2O to remove the free linker and $NaHCO_3$. Finally, the Pyr-S, Amide, Amide-S, and Mal-S (High and Low) were characterized by the DLS in terms of size, PDI, and zeta potentials.

To prepare the biotin-modified Pyr-S, Amide, Amide-S, and Mal-S (High and Low), 1 mg NPs were first incubated with different amount of biotin-NHS (MedChemExpress EU, 0.088, 0.439, and 4.392 µg biotin for low, middle and high degree of modification) in 0.1 M $NaHCO_3$ solution for 30 mins. Then, NPs conjugated with 0.088 µg biotin were further reacted with different linkers (SPDP, DSS, DTSSP, and BMPS) as above-mentioned description.

4. Cell isolation, identification, and culture

Cell isolation: The mice splenocytes were isolated from C57BL/6 mice under the sterile

conditions.[43] After euthanasia, the spleen was removed gently smashed and filtered with 70µm cell strainer (Corning), followed by centrifugating at 400g for 5 mins. Then the cell pellet was treated with red blood lysis buffer (Biolegend) to remove the red blood cells. Finally, the cells were centrifugated (400g, 5 mins) and washed with 1×PBS to obtain the splenocytes.

Cell identification: The splenocytes were stained with APC anti-mouse CD3 antibody (Biolegend) and PE-Cy7 anti-mouse CD8 antibody (Biolegend), followed by analyzing with the flow cytometry (BD, LSRFortessa) to explore the percentage of T cells and cytotoxic T cels in splenocytes.

Cell culture: The isolated splenocytes were cultured in RPMI1640 supplemented with 10% FBS and 1% PS (Penicillin-Streptomycin), and used within 24 hours.

5. The biocompatibility on NPs

To check the biocompatibility of NPs, 0, 2, 4, 10, 40, 100, 200 µg NPs were respectively incubated with 1×10^6 splenocytes for 1 and 3 days in 96-wells plates (200µL/well). At each time point, the plate was centrifugated (800g, 5 mins) and the suspension was discarded. The cell pellet was resuspended with cell titer glo (Promega), according to the instruction. Finally, the luminescent signal was detected by the Varioskan. The cell viability was calculated by the following formulation:

$$Cell\ viability\ (\%) = \frac{\text{Luminescence}_{experimental\ groups}}{\text{Luminescence}_{Control\ groups}} \times 100\%$$

6. Construction and investigation of NCCs.

NPs and cell ratio optimization: NPs (0, 10, 15, 20, 50 µg) were incubated with 1×10^6 splenocytes in 100 µL 1×HBSS (HyClone) at 37 °C for 1 hour to construct the NCCs. After the synthesis, the NCCs were centrifugated (800 g, 5 mins) and washed with 1×PBS to remove the unbinding NPs. The FI of NCCs were quantified by the Varioskan.

NCCs with different linkers: 15 µg Pyr-S, Amide, Amide-S, and Mal-S (High and Low) were respectively incubated with 1×10^6 splenocytes in 100 µL 1×HBSS for 1 hour at 37 °C to construct the NCCs. Subsequently, the NCCs were centrifugated (800g, 5 mins) and washed with 1×PBS to remove the unbinding NPs. Then, the NCCs were characterized by the flow cytometry to evaluate the conjugating efficacy of different linkers. Furthermore, the NCCs were cultured for 1 and 3 days in RPMI1640 supplemented with 10% FBS and 1% PS. At each time point, the NCCs were centrifugated (800g, 5 mins) and washed with 1×PBS to remove the free NPs. Finally, the NCCs were analyzed by the flow cytometry to check the stability in vitro.

Biotinylated NCCs and streptavidin staining: 15 µg biotin-labeled Pyr-S, Amide, Amide-S, and Mal-S (High and Low) were respectively incubated splenocytes to fabricate biotinylated NCCs following the abovementioned protocol. The NCCs right after fabrication (day 0) or after 3 days culture (day 3) was washed and incubated with AF488-labeled streptavidin (Lumiprobe, USA) for 1 hour at 25 °C. Finally, the NCCs were analyzed by the flow cytometry.

7. Statistical analysis

All statistical analyses were carried out using Origin software. As described in figure captions,

one-way ANOVA with Tukey's HSD (honestly significant difference) test were used to determine significance. The n and P values are indicated in the legends. Flow cytometry analyses were carried out using FlowJo V10.

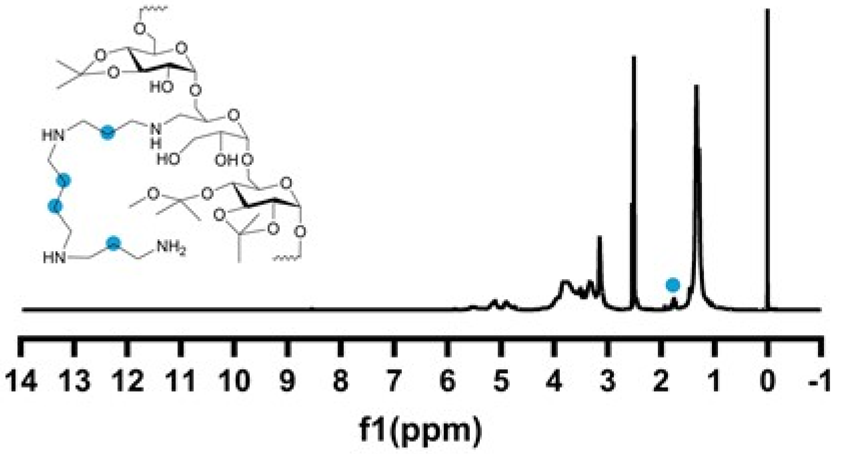

Fig S1. The H-NMR result of Spermine-AcDex (in DMSO-d_6).

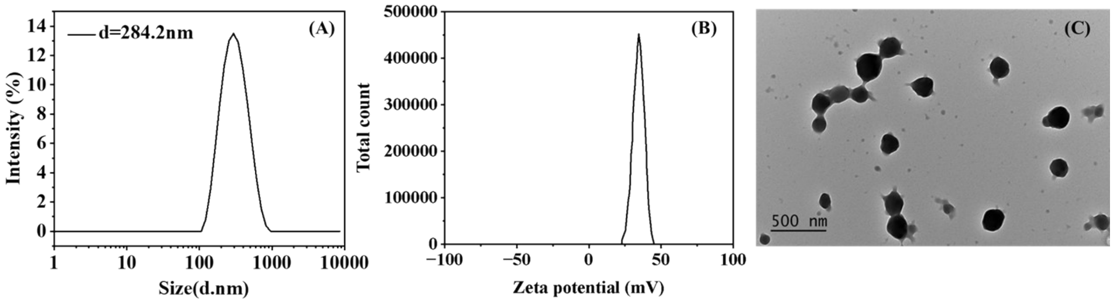

Fig S2. The NPs dynamic light scattering histogram (A) and zeta potential histogram (B), and (C) TEM image.

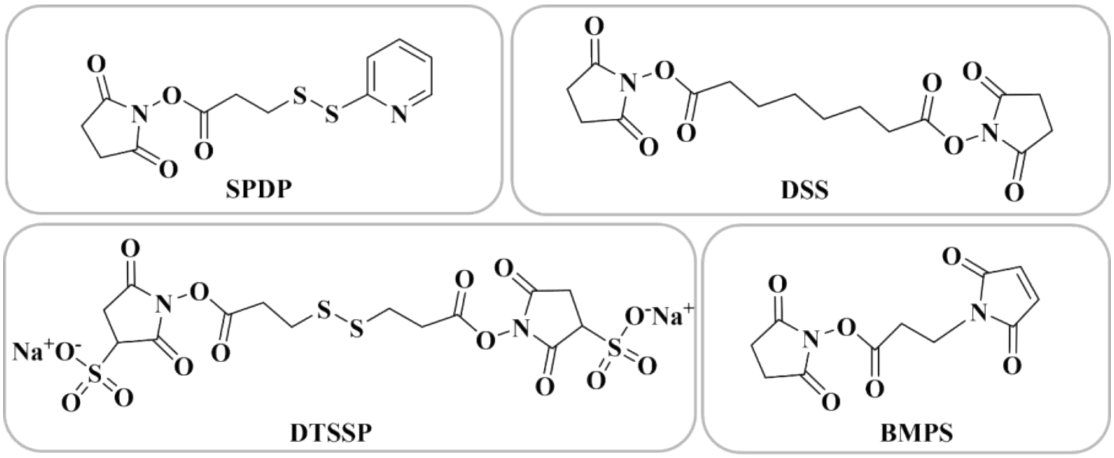

Fig S3. The chemical structures of four different linkers.

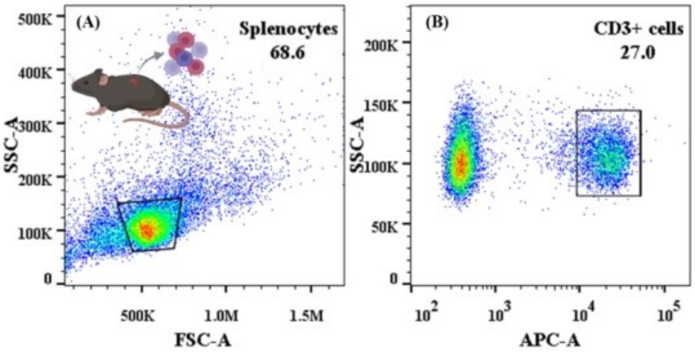

Fig S4. The flow cytometry result of (A) splenocytes, (B) $CD3^+$ T cells, and (C) cytotoxic T cell biomarker (CD8) staining.

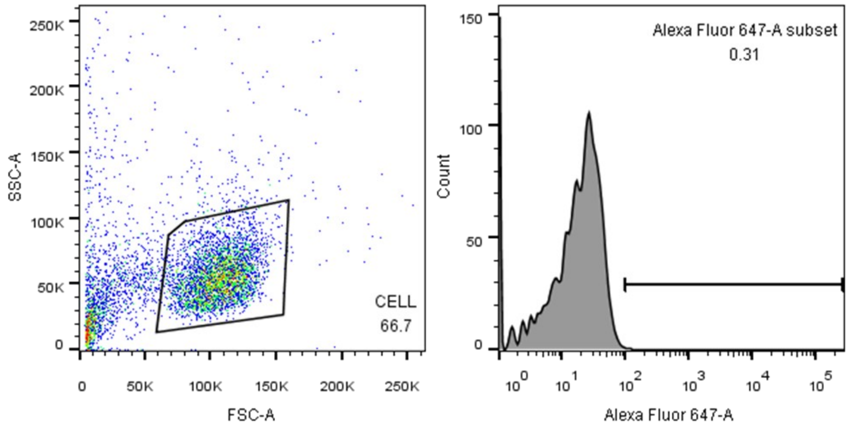

Fig S5. The gating strategy and definition of Alexa Fluor 647–positive events using the negative control sample. (cells alone without NPs).

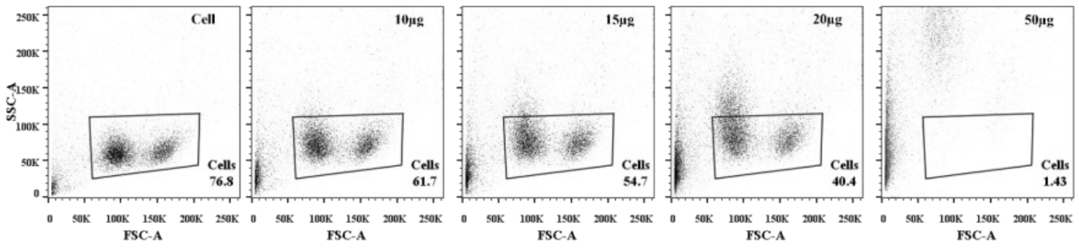

Fig S6. The cell population of NCCs grafting with different amount of NPs.

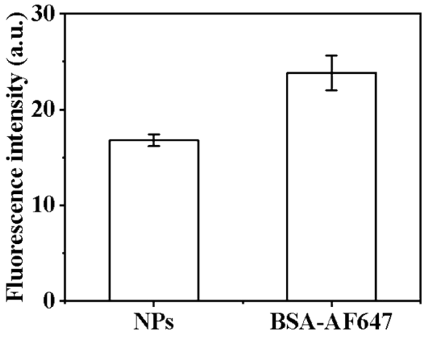

Fig S7. The fluorescence intensity of free Alexa647-labelled BSA (denoted as BSA-AF647) compared to the same amount of BSA-AF647 encapsulated in nanoparticles (denoted as NPs).

Table S1. The elemental analysis of Spermine-AcDex.

N (%)	C (%)	H (%)	S (%)
0.91±0.02	53.62±0.09	8.05±0.07	0.03±0.01
